# Factors Related to Relapse After 6 Months of Smoking Cessation Among Men in the Republic of Korea: A Cross-Sectional Study

**DOI:** 10.1097/MD.0000000000001180

**Published:** 2015-07-24

**Authors:** Eun Young Park, Min Kyung Lim, Byung-Mi Kim, Bo Yoon Jeong, Jin-Kyoung Oh, E. Hwa Yun

**Affiliations:** From the National Cancer Control Institute; National Cancer Center, Goyang, Republic of Korea (EYP, BYJ), Graduate School of Cancer Science & Policy and National Cancer Control Institute; National Cancer Center, Goyang, Republic of Korea (MKL, JKO, EHY), Department of Preventive Medicine, School of Medicine; Ewha Medical Research Center; Ewha Womans University, Seoul, Republic of Korea (BMK).

## Abstract

We identified factors associated with relapse after 6 months of smoking cessation (late relapse) among males of the Republic of Korea. Of the 222,707 smokers who visited public health center-based smoking cessation clinics (SCCs) between January 1, 2009 and mid-December 2009, we included 1720 individuals who successfully completed a 6-month smoking cessation program at an SCC. These participants were selected via a random stratified sampling design and completed an SCC user satisfaction survey between December 31, 2009 and January 6, 2010. Multiple logistic regression was used to identify factors associated with late relapse, and path analysis was employed to explore relationships among these factors. The frequency of late relapse was 21.6% (n = 372). Residence in a metropolitan area, low socioeconomic status, and the use of nicotine replacement therapy (NRT) were associated with statistically significant increases in late relapse, whereas greater access to counseling and more satisfaction with the SCC were associated with reduced late relapse. The path analysis showed that a greater number of cigarettes smoked daily and a younger age at smoking initiation exerted significant indirect effects on late relapse when NRT was employed. Residence in a metropolitan area indirectly prevented late relapse as counseling frequency increased. NRT use, counseling frequency, and SCC user satisfaction were affected by both smoking behavior and socioeconomic status. Relapse prevention efforts should concentrate on increasing both counseling frequency and SCC user satisfaction. Future studies should focus on the effect of NRT on the maintenance of long-term cessation at the population level in real-world settings.

## INTRODUCTION

Tobacco use is the leading cause of preventable death and kills more than 5 million people throughout the world each year. If current trends persist, this figure will increase to more than 8 million by 2030.^1^ Many behavioral and pharmacological interventions have been developed and implemented throughout the world to help people quit smoking, and their effectiveness has been reported in several studies.^2–4^ Although such interventions have been associated with admirable short-term smoking-cessation rates, many who complete smoking-cessation programs relapse within a few weeks.^5^

Thus, postcessation relapse is a critical problem, and factors associated with relapse must be understood if effective relapse-prevention programs are to be developed. Previous studies showed that age, sex, socioeconomic status, and smoking history and behavior were linked to smoking-cessation outcomes. Older smokers quit more successfully than younger smokers,^6,7^ females quit less successfully than males,^8^ those of lower socioeconomic status quit less successfully than those of higher economic status,^7,9^ those who started smoking earlier in life quit less successfully than those who started later, and heavy smokers quit less successfully than lighter smokers. However, most data were obtained after a short-term follow-up period from those who completed smoking-cessation programs. What factors affect late relapse (ie, during long-term follow-up periods) of those who quit after engaging in smoking-cessation programs? Such data would more accurately reflect the success of a program in terms of the ultimate goal: reduction in tobacco use.

Commencing in 2004, Korea has operated public health center-based smoking cessation clinics (SCCs), which operate a nationwide smoking-cessation program funded by the government. The 6-month program features free behavioral counseling and nicotine replacement therapy (NRT). Program effectiveness (the smoking-cessation rate) was earlier explored, and the 4-week smoking-cessation rate was 78%,^2^ which was higher than the equivalent rate of the UK Stop Smoking Services (53%).^10^ However, the long-term outcomes of programs operated by the SCCs and the factors affecting such rates have not been identified. Such data are necessary to improve program content and evaluate program effectiveness.

Thus, we sought to identify factors associated with relapse after 6 months of smoking cessation (late relapse) among Korean males who had successfully completed an SCC smoking cessation program using SCC data and those of an SCC user satisfaction survey.

## METHODS

### Public Health Center-Based SCCs in Korea

The Korean Ministry for Health and Welfare instructed public health center-based SCCs to initiate a 3-month pilot trial in 2004; this was successful, and full operations commenced in 2005. Korea has 253 such SCCs, and all administrative districts are serviced. Each clinic employs a few administrators and doctors, as well as between 1 and 4 trained counselors, depending on the size of the community.

The 6-month smoking-cessation program features systematic, comprehensive behavioral counseling, and enrolment is voluntary. Smokers present at the SCC and are asked to complete a structured questionnaire via a face-to-face interview with a counselor; the questionnaire explores demographic characteristics, smoking habits, and related health risk factors. Nicotine dependence is evaluated using the Fagerström Test for Nicotine Dependence, and physical evidence of smoking status is collected via measurement of carbon monoxide levels and/or urine cotinine testing. All data are recorded electronically.

Next, the smoker sets a quitting date, and free NRT is provided, if necessary. At least 3 face-to-face counseling sessions follow, with additional telephone and texting contact over the next 6 months. Self-reported cessation of maintenance is recorded in program weeks 4 and 6, and at 6 months (when the program ends). If a client reports continued cessation, she or he is categorized as a successful quitter, and SCC services are discontinued. A SCC user who relapses or leaves the program for any reason may re-enrol.

### Study Sample

A total of 222,707 smokers visited all SCCs between January 1, 2009 and mid-December 2009. Of these, 4001 (94%, n = 3783 males; 6%, n = 218 females) were chosen using random sampling by area of residence and program status (ie, discontinued, ongoing, and completed) to participate in an SCC user satisfaction survey. The survey was conducted via computer-assisted telephone interviews conducted between December 31, 2009 and January 6, 2010. The sample was stratified to ensure that the proportion of participants in each selected stratum matched that in the target population. Nonrespondents were replaced by others who were similar in terms of residential area and status in the smoking-cessation program. A total of 1800 responders (n = 4001) who successfully completed the program (ie, who maintained cessation throughout the entire 6 months of the program) were included in analyses seeking to identify factors associated with relapse after 6 months of cessation. Finally, we analyzed data from only the 1720 male respondents only, as few females were included in the sample (n = 80).

### Ethics Statement

The need for written informed consent was waived by the Institutional Review Board of the National Cancer Center of Korea, which approved the study protocol.

### Measures

Baseline data collected at SCC program enrolment included sex; age (20–29, 30–39, 40–49, 50–59, ≥60 years), body mass index (body mass divided by the square of the body height [m], grouped as <23, 23–25, ≥25 kg/m^2^; WHO Expert Consultation),^11^ area of residence (metropolitan, other [small city or country]), type of medical insurance (Medical Aid, National Health Insurance), level of nicotine dependence (Fagerström Test for Nicotine Dependence score: <3 [mild], 4–6 [moderate], 7–10 [severe]), age at smoking initiation (<20 and ≥20 years), duration of smoking (years), cessation support (not supported, friends or other peers, parents or other family members), motivation to contact the SCC (recommendation of another, personal decision), reason for wanting to quit smoking (present, absent), and number of past attempts to quit smoking.

The SCC user satisfaction survey was a structured questionnaire exploring overall satisfaction and satisfaction in terms of certain subcategories, including facilities, registration process and waiting time, SCC coaching and the nature of counseling, cessation aids, and cessation maintenance activities. SCC user satisfaction was rated on a 5-point Likert scale on which 1 meant very dissatisfied, 2 meant dissatisfied, 3 meant neutral, 4 meant satisfied, and 5 meant very satisfied. Subjects were next divided into 2 groups: dissatisfied (very dissatisfied, dissatisfied, and neutral), and satisfied (satisfied and very satisfied). The survey also assessed smoking status after completion of the cessation program with the following questions: “Do you currently smoke?”; “If you have failed to quit, has the number of cigarettes you smoke decreased since completing the program?”; “If you have failed to quit, what was the biggest difficulty you encountered?”

### Data Analysis

The basic characteristics of the study sample, smoking cessation status, and reasons for late relapse (when given) are presented as frequency distributions and were analyzed using the Cochran–Mantel–Haenszel *χ*^2^ test. Multiple logistic regression was used to identify factors associated with late relapse. All statistical tests were conducted with the aid of SAS software (version 9.1.3 Service Pack 3, 2002–2003, SAS Institute Inc, Cary, NC), and *P* < 0.05 was considered to reflect statistical significance.

Path analysis, a form of structural equation modeling, was performed with the aid of Amos software (version 18.0, SPSS Inc, Chicago, IL) to identify factors associated with late relapse in Korean males. The model investigated both direct effects and the effects of individual factors on the behavioral pathways culminating in late relapse. We identified important predictors of smoking cessation based on descriptive analyses identifying factors that were significantly associated with late relapse. Such predictors were identified via backward elimination with the *P* value set at 0.20. The model included baseline data on age, area of residence, extent of nicotine dependence, age at initiation of smoking, and average number of cigarettes smoked per day. The following intermediate variables were also included: NRT use, counseling frequency, and overall SCC user satisfaction (indicators of program quality). To compute path coefficients, a maximum likelihood estimation was performed using a variance/covariance matrix. All coefficients were standardized to allow direct comparisons. To evaluate model fit, we used the *χ*^2^ test to determine the goodness-of-fit-index (GFI) and the root mean square error of approximation (RMSEA). The criteria for good fit were that the *χ*^2^ statistic was nonsignificant (*P* > 0.05), the GFI was greater than 0.90, the RMSEA was less than 0.03, and the comparative fit index (CFI) was greater than 0.90.

## RESULTS

The demographic characteristics (with odds ratios [ORs]) of those who did and did not report late relapse are shown in Table 1. Of all 1720 subjects, 21.6% (n = 372) reported late relapse. Subjects residing in metropolitan areas were more likely to experience late relapse than others (OR 1.59, 95% CI [1.25–2.04]). Data on type of health insurance, which was used as an indicator of socioeconomic status, showed that participants with National Health Insurance were less likely to experience late relapse than those who had Medical Aid (OR 0.55, 95% CI [0.30–0.99]). Participants who used NRT were more likely to experience late relapse than those who did not (OR 2.01, 95% CI [1.50–2.68]). An increased counseling frequency reduced late relapse (OR 0.28, 95% CI [0.19–0.40]). Subjects who were generally satisfied with the SCC program were less likely to experience late relapse than those who were not satisfied (OR 0.51, 95% CI [0.36–0.72]). We found no association between body mass index, extent of nicotine dependence, age at commencement of smoking, or average number of cigarettes smoked per day and late relapse (Table 1).

**TABLE 1:**
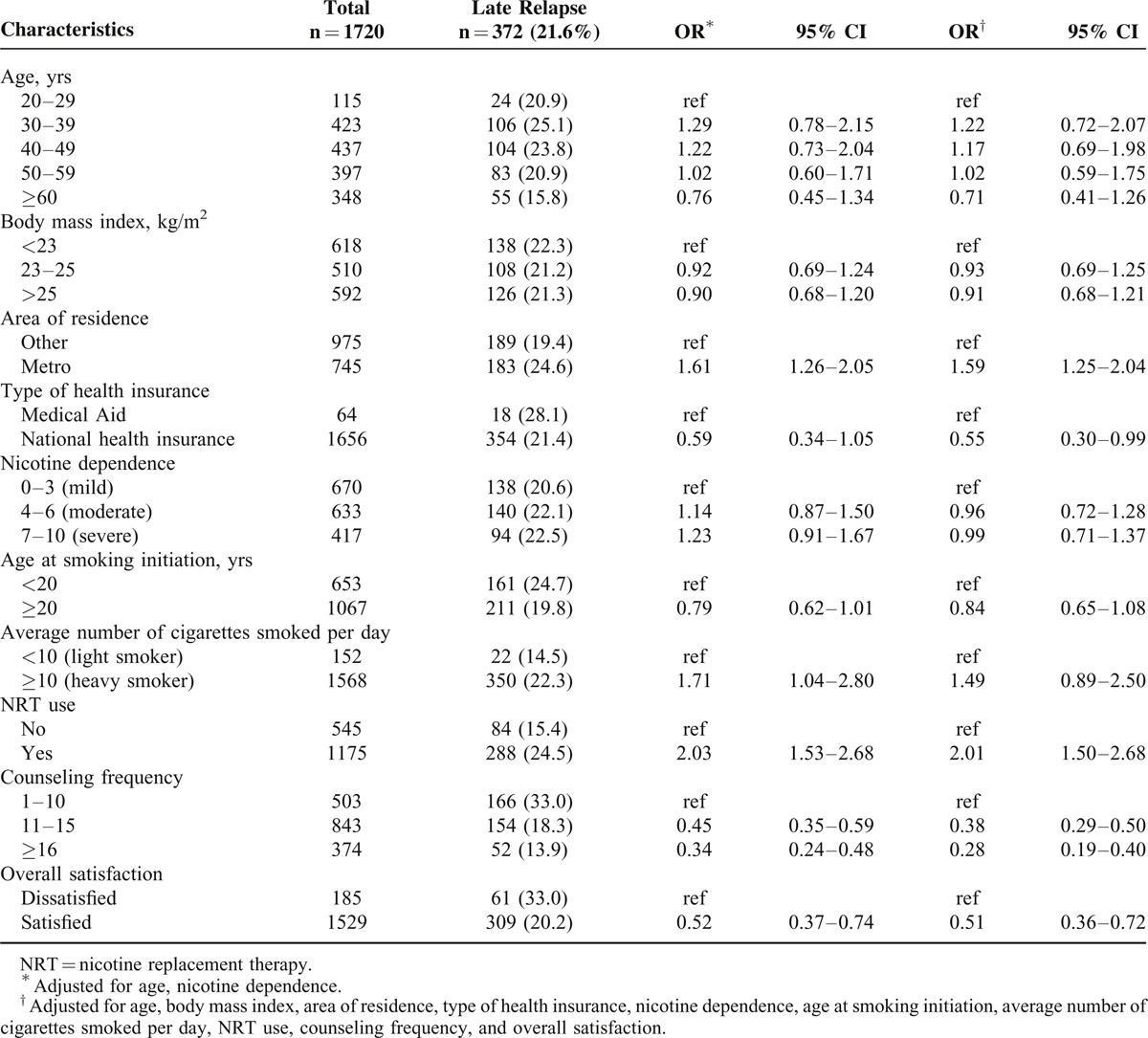
Demographic Characteristics and Odds Ratios (OR) with 95% Confidence Intervals (CI) Comparing Subjects with and without Late Relapse (ie, Relapse After 6 months of Smoking Cessation)

The path analysis model investigated the effects of age, area of residence, type of health insurance, extent of nicotine dependence, age at initiation of smoking, and average number of cigarettes smoked per day at baseline on late relapse, both directly and via possible pathways mediating NRT use, counseling frequency, and overall satisfaction. Although the *χ*^2^ value was high (*χ*^2^ = 8.36, *P* = 0.50), several other indices showed that the model fit was good: RMSEA = 0.001 (≤0.03), GFI = 0.99 (≥0.90), and CFI = 1.00 (≥0.90).

**FIGURE 1:**
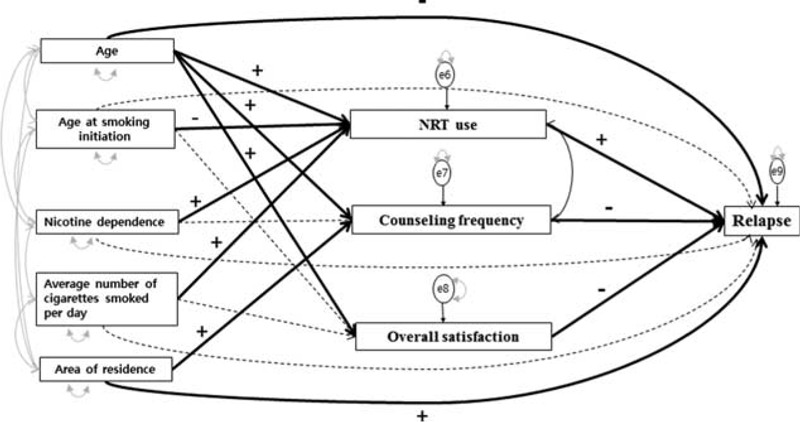
Path diagram for the final model and goodness-of-fit indices. Goodness-of-fit index (GFI) = 0.99, comparative fit index (CFI) = 1, root mean square error of approximation (RMSEA) <0.001, and *χ*^2^ = 8.36 (df 9), *P* = 0.50. The English in this document has been checked by at least 2 professional editors, both native speakers of English. For a certificate, please see: http://www.textcheck.com/certificate/4pmrnV.

The effect of age on late relapse was partially mediated by NRT use (*β* = 0.11, *P* < 0.001), counseling frequency (*β* = 0.08, *P* < 0.001), and overall satisfaction with the program (*β* = 0.07, *P* = 0.003) (Figure 1, Table 2). Age was also directly associated with late relapse (*β* = −0.05, *P* = 0.03). The effects of age at smoking initiation (*β* = −0.07, *P* < 0.006), average number of cigarettes smoked per day (*β* = 0.20, *P* < 0.001), and extent of nicotine dependence (*β* = 0.09, *P* < 0.001) on late relapse were partially mediated by NRT use. In addition, the effect of area of residence on late relapse was partially mediated by counseling frequency (*β* = 0.12, *P* < 0.001) and was also directly associated with late relapse (*β* = 0.08, *P* < 0.001). Thus, the average number of cigarettes smoked per day had a larger impact on NRT use than did other variables. Counseling frequency (*β* = −0.19, *P* < 0.001) and NRT use (*β* = 0.12, *P* < 0.001) had major direct effects on late relapse (Table 2).

**TABLE 2:**
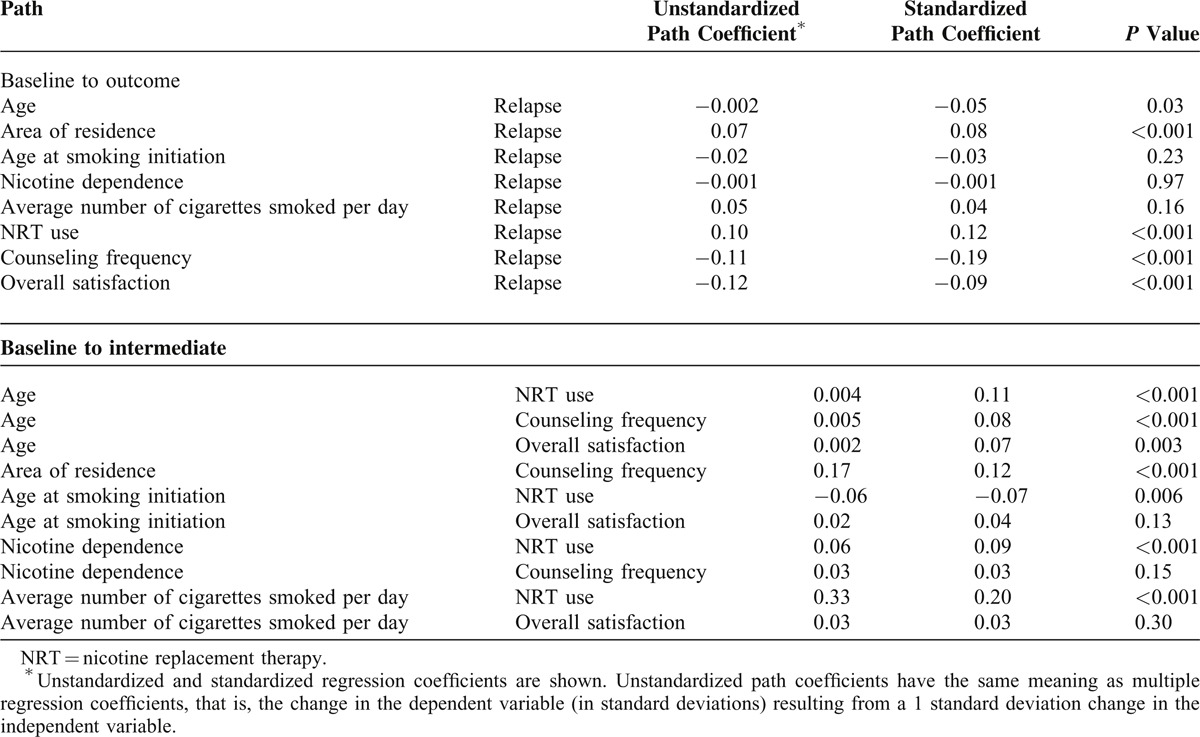
Path Coefficients from the Path Analysis

Table 3 shows the total effects of each path according to whether its effect was direct and indirect. Age (*β* = −0.06, *P* = 0.02), area of residence (*β* = 0.06, *P* = 0.02), and average number of cigarettes smoked per day (*β* = −0.06, *P* = 0.05) exerted the most powerful total effects. Area of residence, age at smoking initiation, and average number of cigarettes smoked per day exerted statistically significant indirect effects on late relapse: *β* = −0.02, *β* = −0.01, and *β* = 0.02, respectively (*P* = 0.01 for all comparisons). Age and area of residence exerted significant direct effects on late relapse: *β* = −0.05 and *β* = 0.08, respectively (*P* = 0.03 and *P* = 0.02, respectively) (Table 3).

**TABLE 3:**
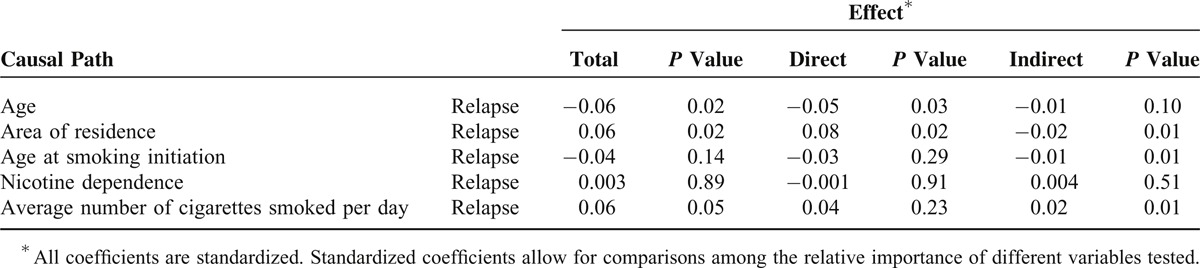
Total, Direct and Indirect Effects of Independent Variables on Dependent Variables by Path Analysis

## DISCUSSION

We identify, for the first time, factors associated with late relapse among Korean males. The effect of NRT on the maintenance of long-term cessation was not as positive as previously thought,^12,13^ and NRT may actually adversely affect long-term cessation. The extent of SCC user satisfaction independently affected cessation maintenance, emphasizing the need to consistently reinforce smoking cessation via user feedback. Finally, the social environment of participants seemed to play an important, albeit complex, role in the long-term cessation of smoking. Residents of metropolitan areas participated in counseling more frequently, which indirectly maintained cessation, but living in a metropolitan area also had the direct effect of increasing late relapse. Lower socioeconomic status also seemed to encourage late relapse, which is consistent with data from previous studies.^7,9^

One unexpected (and significant) finding was that NRT users were more likely to experience late relapse; this finding emerged not only from multiple logistic regression analyses but also from path analyses, even when confounding factors were controlled. Although NRT use did not directly affect late relapse, the more cigarettes smoked per day and the earlier the age at smoking initiation, the more NRT use negatively affected late relapse. These results are not in agreement with those of previous studies, which emphasized the positive effects of NRT in terms of cessation and relapse prevention.^12,13^ However, although the short-term efficacy of NRT has been well documented, scarce data on the effects of NRT on maintenance of long-term cessation are available.^14^ In addition, smoking-cessation medications, including NRT, are cause for concern. Currently, no population-based evidence that medications improve smoking-cessation rates is available, which is possibly attributable to the low level of effectiveness of such medications in community settings.^15,16^ In reality, despite increased NRT use, smoking has not declined in Korea, implying that most smoking-cessation attempts are unsuccessful. It is possible that SCC users did not employ NRT appropriately, thus reducing any effect thereof.^17^

Another finding of note was that, overall, SCC user satisfaction was very high and that such satisfaction was significantly associated with the maintenance of cessation. Currently, little is known about associations between the satisfaction of those who use clinics and smoking-cessation outcomes. However, this finding is consistent with that of our previous study employing data from the Korean Quit-line program on the effect of user satisfaction on smoking-cessation outcomes^18^ and with the conclusion of another study.^19^ Thus, user satisfaction is important for the prevention of relapse, and it is important to continue to improve smoking-cessation programs based on user feedback.

We also found that the social environment and socioeconomic status (area of residence and type of health insurance) affected late relapse. Residents of metropolitan areas participated in counseling more frequently because they were able to access SCCs more easily and were also more frequently exposed to cessation campaigns and anti-smoking education, which reduced late relapse. Thus, program accessibility is important. However, when both the direct and indirect effects (mediated by counseling frequency) influencing relapse were considered, living in a metropolitan area actually increased late relapse. This may be because life is more complex and stressful in metropolitan areas than elsewhere. In Korea, smoking and drinking have been considered acceptable means by which stress caused by complex social rules can be relieved; this attitude continues to influence the environment and culture in big cities. Furthermore, ex-smokers are often accustomed to smoker-friendly environments (such as bars), which may increase the urge to smoke and contribute to late relapse. In the present study, Medical Aid recipients were more likely to relapse than were those covered by National Health Insurance; this is consistent with data from previous studies showing that subjects of lower socioeconomic status (with less education and low incomes) were more likely to smoke and fail to quit smoking.^7,9^

Although our findings are novel, our study has several limitations. First, the research was retrospective in nature, and it is thus unclear whether greater satisfaction with SCCs triggered successful cessation or whether the converse was the case (ie, successful cessation rendered an SCC user more satisfied). If the latter were true, user satisfaction would be associated with, rather than predictive of, successful smoking cessation. Second, our sample size was relatively small, with few females and younger subjects. Therefore, our sample may not be representative of the general population. However, we sought to minimize this concern by randomly sampling all SCC users and by stratifying the sample by area of residence and status in the smoking-cessation program. However, we included relatively few subjects younger than 30 years of age, and all subjects were male. Otherwise, our sample was similar to the overall population. Additionally, although most smokers who visit public health center-based SCCs are from the general population of the area and do not suffer from any disease that could affect the probability of relapse, we cannot fully exclude the effects of diseases associated with smoking (respiratory and cardiovascular diseases, cancers, periodontitis, etc.)^1,20,21^ on relapse. Thus, some subjects may have had underlying diseases. Finally, as in other healthcare satisfaction surveys, a “courtesy bias” might have led users to provide misleadingly favorable responses. However, such bias may not have greatly influenced the results, as the satisfaction survey was conducted by telephone using trained interviewers employed by an independent external institute.

Smoking cessation interventions, including Quitlines and SCCs, have been implemented in many countries; these measures are cost-effective and play important roles in public health.^3,4,22–24^ However, although the goal of any intervention is to maintain life-long abstinence, most participants relapse within 3 months of a smoking-cessation attempt, and most are smoking cigarettes on a daily basis within 1 year. Thus, relapse prevention is very important. Korean public health center-based SCCs have been shown to effectively and efficiently help smokers quit.^2^ However, the present study suggests that, to encourage maintenance of long-term cessation, higher-quality easy-access smoking cessation intervention services are required. More importantly, we found that NRT aids long-term cessation maintenance less than it aids short-term cessation; thus, in the real world, NRT may not be as useful as previously thought. This needs to be considered when establishing or optimizing smoking-cessation programs.
